# Medical physics aspects of cancer care in the Asia Pacific region: 2011 survey results

**DOI:** 10.2349/biij.8.2.e10

**Published:** 2012-04-01

**Authors:** T Kron, HA Azhari, EO Voon, KY Cheung, P Ravindran, D Soejoko, K Inamura, Y Han, NM Ung, L Bold, UM Win, R Srivastava, J Meyer, S Farrukh, L Rodriguez, M Kuo, JCL Lee, A Kumara, CC Lee, A Krisanachinda, XC Nguyen, KH Ng

**Affiliations:** 1 Physical Sciences, Peter MacCallum Cancer Centre, and RMIT University, Melbourne, Australia; 2 Department of Medical Physics and Biomedical Engineering, Gono Bishwabidyalay (University), Savar, Dhaka, Bangladesh; 3 Radiation Safety and Quality Unit, Department of Scientific Services, Ministry of Health, Brunei Darussalam; 4 Department of Clinical Oncology, Prince of Wales Hospital, Hong Kong, China SAR; 5 Department of Radiation Oncology, Christian Medical College, Vellore, India; 6 Physics Department, University of Indonesia, Jakarta, Indonesia; 7 Professor Emeritus, Osaka University, Onoharahigashi 6-35-7, Minoh-city, Japan; 8 Sungkyunkwan University, Samsung Medical Centre, Republic of Korea; 9 Clinical Oncology Unit, University of Malaya, Kuala Lumpur, Malaysia; 10 Radiotherapy Department, National Cancer Centre, Ulaanbaatar, Mongolia; 11 Ministry of Health, Yangoon, Myanmar; 12 B.P. Koirala Memorial Cancer Hospital, Bharatpur, Chitwan, Nepal; 13 Department of Physics and Astronomy, University of Canterbury, Christchurch, New Zealand; 14 Department of Radiation Oncology, Atomic Energy Medical Centre, Karachi, Pakistan; 15 Department of Radiotherapy, Jose R. Reyes Memorial Medical Center, Manila, Philippines; 16 Department of Radiation Oncology, Cancer Institute (Hospital), Chinese Academy of Medical Sciences; 17 Department of Therapeutic Radiology, National Cancer Centre, Singapore; 18 Division of Medical Physics, National Cancer Institute, Sri Lanka; 19 Department of Medical Imaging and Radiological Sciences, Chang Gung University, Taiwan; 20 Department of Radiology, Faculty of Medicine, Chulalongkorn University, Bangkok, Thailand; 21 K Hospital, National Cancer Institute, Hanoi, Vietnam; 22 Department of Biomedical Imaging, University of Malaya, and Medical Physics Unit, University of Malaya Medical Centre, Kuala Lumpur, Malaysia

## Abstract

**Background:**

Medical physicists are essential members of the radiation oncology team. Given the increasing complexity of radiotherapy delivery, it is important to ensure adequate training and staffing. The aim of the present study was to update a similar survey from 2008 and assess the situation of medical physicists in the large and diverse Asia Pacific region.

**Methods:**

Between March and July 2011, a survey on profession and practice of radiation oncology medical physicists (ROMPs) in the Asia Pacific region was performed. The survey was sent to senior physicists in 22 countries. Replies were received from countries that collectively represent more than half of the world’s population. The survey questions explored five areas: education, staffing, work patterns including research and teaching, resources available, and job satisfaction.

**Results and discussion:**

Compared to a data from a similar survey conducted three years ago, the number of medical physicists in participating countries increased by 29% on average. This increase is similar to the increase in the number of linear accelerators, showing that previously identified staff shortages have yet to be substantially addressed. This is also highlighted by the fact that most ROMPs are expected to work overtime often and without adequate compensation. While job satisfaction has stayed similar compared to the previous survey, expectations for education and training have increased somewhat. This is in line with a trend towards certification of ROMPs.

**Conclusion:**

As organisations such as the International Labour Organization (ILO) start to recognise medical physics as a profession, it is evident that despite some encouraging signs there is still a lot of work required towards establishing an adequately trained and resourced medical physics workforce in the Asia Pacific region.

## Introduction

The International Labour Organization (ILO) has recently classified ‘medical physicist’ as an occupation in the International Standard Classification of Occupations-08 (ICSO-08) under physicists and astronomers [1]. This is indicative of the increasing awareness of medical physics as a profession in its own right. While the actual number of medical physicists is relatively small compared to other professional groups in medicine, they perform a vast variety of different tasks. This applies also to its subspecialty of radiation oncology medical physics, in which the majority of medical physicists work.

In 2008, a survey was conducted to determine the characteristics of the medical physics workforce in countries within the region of the Asia-Oceania Federation of Organizations for Medical Physics (AFOMP) [2]. It probed workforce numbers and typical workloads, training and education, and job satisfaction among medical physicists working in radiation oncology in 16 countries in the Asia Pacific region. The aim of the present study was to update this information three years after the initial survey. In addition to this, questions on certification and research opportunities were added to enrich the data and reflect on recent trends in health professions.

As in 2008, the definition of the clinical medical physicist used was that of the AFOMP [3]. It is worth repeating here:

“A qualified Clinical Medical Physicist is a person who is qualified with a master university degree or equivalent in physical science or engineering science and working in alliance with medical staff in hospitals, universities or research institutes. In addition to his/her university degree or equivalent, a Clinical Medical Physicist shall have specialist training in the concepts and techniques of applying physics in medicine including training in the medical application of both ionizing and non-ionizing radiation. This person must have a thorough knowledge in one or more sub-fields of medical physics, including radiotherapy physics, imaging physics, nuclear medicine physics and radiation protection.”

A key point in this definition is that a medical physicist has both a sound theoretical understanding of the science as well as clinical training in its application. As technology advances rapidly, an additional requirement is commonly the participation in Continued Professional Development (CPD) [4, 5]. This requires significant resources that may not be available everywhere. There is therefore an important role for professional organisations such as the AFOMP, which has the mission to advance and standardise medical physics practice in the region. In this context, AFOMP has initiated a periodic survey of medical physics practice in the region, the first of which was conducted in 2008. The present report summarises the outcomes of the second survey in 2011.

Specifically, it is the aim of the present article to:

- Document differences and commonalities in education and clinical experience required for radiation oncology medical physicists (ROMPs) in the Asia Pacific region,
- Update general information on the number of physicists and their relation to equipment and other professions,
- Document the tasks undertaken by physicists in radiation oncology, including research and teaching, and
- Explore resources, status and job satisfaction available to and among medical physicists.

Where possible, comparisons with the 2008 survey have been included to highlight any changes over the three-year period.

## Methods

Based on the 2008 survey [2], a questionnaire was designed to explore medical physics practice in radiation oncology. The 2008 survey relied on an ad-hoc group of medical physicists in 16 countries to provide information on the situation in these countries. As this approach was successful, a similar approach was used in 2011.

The questionnaire was sent to 20 senior physicists in the region who have been active in the field for several years. Many of them have represented their medical physics organisations at AFOMP, the International Organization of Medical Physics (IOMP), and the International Atomic Energy Agency (IAEA) and were, as such, considered to be familiar with the state of medical physics in their respective countries. Including the initiators of the survey, the questionnaires reached 22 countries and territories in the Asia Pacific region.

The questionnaire was distributed in English and covered seven main areas in five themes with all questions asked in 2008 included (some with minor clarifications). Some additional questions were included to probe important areas further.

1. Education, training and professional certification

In addition to questions on expected training, education and CPD, the questionnaire probed the availability of a certification scheme.

2. Staffing numbers and treatment equipment

This section also included an assessment of the ratio of ROMPs to other professions and the overall population in participating countries. Most updated information on population numbers was taken from Wikipedia (en.wikipedia.org). It reflects data from 2010 or 2011 in all cases. Information on equipment provided in the questionnaires was supplemented with data from the Directory of Radiotherapy Centres (DIRAC) database maintained by the International Atomic Energy Agency (IAEA) (http://nucleus.iaea.org/HHW/DBStatistics/DIRAC/index.html).

3. Workload

This part of the questionnaire was enlarged compared to 2008 to provide more details on the typical activities of ROMPs in the region. For example, information technology (IT) was explicitly included and we also inquired about the requirement for ROMPs to work overtime.

4. Professional organisations

5. Resources available

6. Research and teaching

7. Job satisfaction in the areas of professional recognition, remuneration, and workload.

In addition to this questionnaire, participants were invited to provide as many free form comments as necessary. The questionnaire was sent out by email in March 2011 and the original time frame for answering the questions was four weeks; however, answers were accepted beyond the four-week period. They reflect the status of March to August 2011. On some occasions, additional details were elicited and provided in communication with participants.

## Results

Answers were received from all 22 countries/territories, representing more than 3500 medical physicists. This constitutes a response rate of 100% compared to 80% in 2008 [2].

Tables 1 to 5 show the results. The numbering and structure of the tables is identical to that used in the original publication [2] to facilitate easy comparisons. Two tables (2 ‘staff numbers and equipment’ and 3 ‘workload’) were split into two tables each to make access to the data easier. Table 2 also contains a direct contrasting of figures from 2008 and 2011.

**Table 1:** Education and training of radiation oncology medical physicists (ROMPs) in the Asia/Pacific region.

	**What is the expected academic degree for ROMPs?**	**Is there a formal training program?**	**Typical length of clinical training (years)**	**Is there professional certification?**
Australia	MSc	yes	3	yes, by ACPSEM
Bangladesh	MSc	under development	2 (planned)	planned
Brunei	MSc preferred	no		no
Hong Kong China	MSc, PhD required for higher rank	yes, residency program	2	yes, since 2006
India	MSc	yes, residency available at selected hospitals	1	yes, by CMPI
Indonesia	MSc		2 (planned)	
Japan	BSc required	in development	3 years clinical experience	yes by Japanese Board of Medical Physicists
Republic of Korea	MSc	yes, two programmes accredited	3 (2 if PhD)	yes includes a 100hrs lecture course by KSMP
Malaysia	BSc, MSc preferred	not local but IAEA ROMP training	2 to 3	no
Mongolia	BSc required	in discussion	3 months required	planned within 10 years
Myanmar	MSc	no		no but planned
Nepal	MSc	no		planned
New Zealand	MSc	yes	3	yes, by ACPSEM
Pakistan	MSc	only MS in medical physics	1	planned, expected to commence within next 05 years
Papua New Guinea	not applicable	no		no
Philippines	MSc preferred	participation in IAEA pilot	2 to 3	commenced in 2010
PR China	MSc preferred	no	2 years clinical experience	yes exam
Singapore	MSc preferred	each hospital determines its own	2 + overseas attachment	no - profession too small
Sri Lanka	MSc to be completed within 5 years after selection	yes		planned within 3 years - lack of supervisors
Republic of China (Taiwan)	BSc, MSc preferred - now MSc de facto	3 MS/PhD programs, no residency program	varies from 2 years for PhDs to 5 years for BScs	yes, exam and 1 year CSMPT membership
Thailand	MSc	yes - grad dip of clinical sciences program in Med Phys (2 years)	2 years	yes, exam + clinical training
Vietnam	BSc, MSc preferred	yes but syllabus may depend on hospital		planned within 2 years

**Table 2a:** Number of ROMPs and irradiation equipment in the Asia/Pacific Region.

	**Number of ROMPs**		**Brachytherapy units**	**Tele Cobalt units number**	**Linacs number**	Other*
	**2008**	**2011**				
Australia	224	274	about half the centres HDR, more I125 seeds in urology	0	130	IMRT, IGRT, HT 1, GK 1
Bangladesh	9	23	5 (2 LDR, 3 HDR)	11	8	IMRT 2, IGRT 1
Brunei		2			in the process of tendering	
Hong Kong China	42	43	full range	0	32	HT 3, CK 1, GK 1, IMRT, IGRT
India	550	800	LDR 15, HDR 173	277	157	HT 3, GK 8, CK 2, IMRT, IGRT
Indonesia	38	42	17	17	16	IMRT, IGRT, SRS
Japan		562	LDR 1, HDR 124	11	816	HT 16, GK 46, CK 265, MT 15, P 10, IMRT, IGRT
Republic of Korea	66	81		0	119	CK 8, PT 1, HT 14
Malaysia	55 to 60	80	several (4 from DIRAC)	1	32	CK 1, HT 2, IMRT, IGRT
Mongolia	3	6	1	2	linac will be installed in 2 years	
Myanmar		4	5 (from DIRAC)	6	0	
Nepal	10	10	2	4	3	
New Zealand	44	55	offered in few centres	0	25	IMRT, IGRT
Pakistan		47	11	18	21	IMRT, IGRT, SRS, CK1 GK1
Papua New Guinea		0	manual in the past	1	0	
Philippines	30	40	16 centres with brachytherapy	8	26	10 IMRT, 3 IGRT,4SRS GK 1
PR China	1181	1500	many	500	1200	CK 1, IMRT, IGRT, SRS
Singapore	13	17	full range (3 from DIRAC)	0	18	HT2, GK 1, IMRT, IGRT
Sri Lanka	8	9	3 (from DIRAC)	10	2	
Republic of China (Taiwan)	100	142	46 (all HDR)	4	128	HT 10, CK 5, GK 8, P 1 (under construction), IMRT, IGRT, SRS
Thailand	76	81	24 (from DIRAC)	23	45	CK 1, GK 1
Vietnam	25	50	7 (from DIRAC)	14	17	CK 1, GK 3
**Total**	**2410**	**3864**		**907**	**2798**	

* IMRT = Intensity Modulated Radiation Therapy, IGRT = Image Guided Radiation Therapy, HT = Helical Tomotherapy, CK = Cyberknife, GK = Gammaknife, PT = proton and particle therapy, MT = microtron based radiotherapy, SRS = stereotactic radiosurgery

**Table 2b:** Ratio of ROMPs to other professionals and the population in general.

	**Oncologists/ ROMP ratio**	**Patients/ ROMP ratio**	**MV Machine/ROMP**		**Population (millions)**	**MV Machine/Mn Pop**	
			**2008**	**2011**		**2008**	**2011**
Australia	1.3	300	0.54	0.47	22.7	5.83	5.73
Bangladesh	6.1	8696	1.56	0.956	159	0.09	0.14
Brunei	1.0				0.4		
Hong Kong China	2.0	250	0.79	0.74	7.1	4.71	4.51
India	2.0	300	0.70	0.54	1210	0.34	0.36
Indonesia	1.0	350	0.76	0.79	238	0.13	0.14
Japan	3.4	778		1.47	128		6.46
Republic of Korea	2.5	730	1.55	1.47	49	2.07	2.43
Malaysia	0.6	350		0.41	27.5		1.20
Mongolia	2.0	1000	0.67	0.50	2.8	0.67	0.71
Myanmar	5.0	1500		1.50	48		
Nepal	0.5	450	0.70	0.70	29	0.24	0.24
New Zealand	1.0	250		0.45	4.4		5.68
Pakistan	3.0	1200		0.83	177		0.22
Papua New Guinea					6.7		
Philippines	1.3	400	1.00	0.88	94	0.32	0.38
PR China	4.0	400	1.18	1.13	1339	1.04	1.27
Singapore	2.0	400	1.38	1.06	5.1	3.91	3.53
Sri Lanka	2.0	1500	1.38	1.33	21	0.52	0.57
Republic of China (Taiwan)	1.5	300	1.14	0.93	23	5	5.74
Thailand	1.2	330	0.78	0.84	67	0.9	1.01
Vietnam	1.4		0.92	0.62	87	0.27	0.36
**Total (mean)**	**2.0**	**614.6**	**(mean 0.96)**	**(mean 0.88)**		**(mean 1.9)**	**(mean 2.1)**

**Table 3a:** Workload of ROMPs.

	**Clinical**			**Official working hours**	**Other**			
	**QA, calibration, commissioning**	**Engineering/ maintenance**	**Radiotherapy Treatment Planning**		**Administration**	**Radiation Protection**	**IT**	**Research and teaching**
Australia	10	2	4	38	2	2	4	4
Bangladesh	3	2	6	work hours differ public and private	3	1	1	2
Brunei	no data yet						10	10
Hong Kong China	10	5	12	44	5	4	4	4
India	10	2	16		6	2	1	5
Indonesia		2	15	40		2		8
Japan	13	3	13	40	8	2	10	20
Republic of Korea	12	2	10		10	1		20
Malaysia	10		15		2	2	1	2
Mongolia	1	1	10	35	1	1	1	1
Myanmar	1	3	5		"all of duty time"	2	a few hours	0
Nepal	7		26	42	1	2	0.5	1
New Zealand	15	3	7	38	5	2	5	3
Pakistan	9	2	20	40 to 45	6	2	2	4
Papua New Guinea	no data							
Philippines	3	1	20	40	12	2	2.5	2
PR China	10	6	30	40 to 50	2	1	6	1
Singapore	15	3	15	42	4	1	2	1
Sri Lanka	2	1	30		2	2	1	3
Republic of China (Taiwan)	5	1	28	40	3	1	2	3
Thailand	10	0	20		2	1	2	5
Vietnam	8.5	1	27.5	42 (six days/week)	2.5	1.5	4.5	1.5
**Average**	**8.1**	**2.2**	**16.5**		**4.25**	**1.7**	**3.3**	**4.0**

**Table 3b:** Involvement of ROMPs in research and teaching and the requirement of doing overtime.

	**Research**		**Teaching**		**Overtime**	
	**Participation**	**Clinical trials**	**To physicists**	**To others**	**% of staff working regular working OT**	**Overtime allowance?**
Australia	y	some	y	oncologists, RTs	> 50	often time in lieu
Bangladesh	y	n	y	oncologists (in process)	50	no
Brunei	y	n		diagn radiol	100	no
Hong Kong China	y	y	y	wide variety of teaching tasks	70	no
India	y in teaching hospitals	y in some centres	y	y	>50	some clinics yes
Indonesia						
Japan						
Republic of Korea					most	only junior staff
Malaysia	not all	very few	y	y	50	no
Mongolia	not yet	in planning	in discussion		most	not sufficient
Myanmar					most	no
Nepal	sometimes	sometimes		y RadOncs and BSc Technol	no	no
New Zealand	small percentage	y		RadOncs and RTs	5	yes
Pakistan	y	not in general	y	y	35	2/3 have allowance
Papua New Guinea	not applicable					
Philippines	limited	in planning	y	y	99	some yes, many no
PR China	Participation in research	few		in uni centres - med students	60	no
Singapore	some	some		y RTs and medical officers	30	no
Sri Lanka	few	no		MD oncology, radiol, radiography	most	no, but requested
Republic of China (Taiwan)	y	y	y	y	most	no
Thailand	y	y		y	100	yes
Vietnam	few	not often	y	y	15	yes but not quite satisfactorily

**Table 4:** Professional organizations and access to resources – for explanation of acronyms for the professional organizations, please refer to appendix 1.

	**Professional organisations**			Access to*			**Internet access (%)**
	**Medical Physics**	**Number of members**	**Other relevant organisations**	**Dosimetry equipment**	**literature**	**Discussion with colleagues**	
Australia	ACPSEM	450	ARPS, AAPM, IPEM	e	e	g	100
Bangladesh	BMPS	86	some	a	g	g	90
Brunei	no			g	a	a	100
Hong Kong China	HKAMP	82	40% o/s	e	e	g	100
India	AMPI and brachysociety	1900	yes	e	g	e	99
Indonesia	HFMBI/IKAFMI	48		g	a	a	100
Japan	JSMP	1720	JSRT	e	e	e	>80
Republic of Korea	KSMP	275	yes	g	a, private hospitals may have problems	g	100
Malaysia	MIP/MP subgroup, MAMP	35	yes	g	g	g	99
Mongolia	starting		no	a	g	a	100
Myanmar	National committee through RAS 6053		no	a	n	a	50
Nepal	NAMP	10	y (AAPM)	g	n	a	60
New Zealand	ACPSEM	77	y	a	g	g	100
Pakistan	POMP		PSCO, PSC, RSP, PSNM	a	a	g	90
Papua New Guinea				a	a	n	100
Philippines	POMP	88	PARP	most a, few n	n	g	100
PR China	CSMP	1500	most are members of CSRO; 100 IPEM	n except for top centres	a except for top centres	only in 25% of centres	90
Singapore	SMP (Society of MP)	16	y	e	g	g	100
Sri Lanka	SLMPA	8	n	a	a	a	75
Republic of China (Taiwan)	CSMPT	250	y (CSTRO)	e	e	e	100
Thailand	TMPS	120	Radiol Soc Thailand, AAPM	a	g	g	100
Vietnam	VAMP	450	Viet Soc Cancer	n	a	g	30
**Total**		**7107**					

* Categories: excellent: e, good: g, acceptable: a, not adequate: n

**Table 5:** Job satisfaction of ROMPs and general comments (1 worst, 5 best).

	**Work conditions/job satisfaction**			**Comments**
	**Professional recognition**	**Remuneration**	**Workload (1 = too much, 5 = easy)**	
Australia	4	4	2	variation in states; significant improvement over last years
Bangladesh	3	3	1	more training required; AFOMP, IAEA play important role
Brunei	3	3	2	not sufficient understanding of med phys in administration; radiation does not have high priority'
Hong Kong China	4	3	2	
India	3	3	1	remuneration varies between govt and private
Indonesia				
Japan	1	1	3	most med phys tasks are performed by 'radiological technologists'; they are well trained to do the job but not called medical physicists; eighty percent of medical physicists now also have license of radiological technologist.
Republic of Korea	2	2	2	KSMP is persuing legislation for medical physics
Malaysia	2	3	3	professional recognition needs to be improved; accreditation program needed
Mongolia	5	3	2	developing country cannot afford to participate in o/s training; CPE and training could be regional?
Myanmar	3	2	1	
Nepal	2	3	3	medical physics is new and as such recognition not good; not an IAEA member
New Zealand	2	3	3	
Pakistan	3	4	3	no med phys courses at uni - lack of research culture; most work routine - promotion should come also from outside
Papua New Guinea				no permanent physicist - remote support from Australia
Philippines	4	3	2	ROMP salary not standardised, POMP has no negotiation power; quite a number of med physicists work in regulatory agencies
PR China	2	2	2	status has improved with 3D CRT and IMRT; no professional title as yet - hinders promotion
Singapore	3	3	3	
Sri Lanka	2	1	1	recently 17 physicists with BSc were recruited; training is needed
Republic of China (Taiwan)	4	3	2	
Thailand	4	3	2	
Vietnam	2	2	3	IAEA assistance needed; QA tools - daily - required

As in 2008, about half of the respondents provided additional information in free form. This information was included in the tables wherever possible and additional comments shown are in table 5.

## Discussion

As the profession of medical physicists is maturing and international organisations such as the IAEA and the IOMP are aiming to standardise education and practice, it is of interest to explore how medical physicists fare in the Asia Pacific region. The fast and comprehensive reply of respondents in all countries/territories approached illustrates that this interest is shared by ROMPs. The higher response rate (100% in 2011 compared with 80% in 2008) may reflect increasing professional awareness but could also be a result of allowing more time for completion of the questionnaires.

### Education, training and professional certification

As in 2008, all respondents agreed on the need for a combination of academic education and clinical training. This is in line with developments all over the world. In most countries, the need for postgraduate education specialising in medical physics was also acknowledged. It will be a challenge to ensure that access to these courses is available everywhere. The internet provides a unique opportunity here and web-based resources can at least provide some of the required content [6]. While the need for a PhD [7] is not apparent in the answers, Table 1 shows that several countries give candidates with a PhD advanced standing and reduce requirements for other training. This practice needs to be considered case by case as academic education cannot necessarily replace clinical experience.

There is no doubt that medical physicists working in radiation oncology require a high level of training and specialisation. Taking into account a higher degree and clinical training that typically takes at least two years, entry into the profession typically requires at least seven years of specialist education after high school completion. As such, ROMPs are amongst the most highly trained professionals without a medical degree in hospitals.

It is therefore not surprising to find a trend towards a requirement for professional certification of medical physicists [8, 9]. Patients and other medical professionals find it difficult to judge the competence of ROMPs. As such, they need to rely on peer review and assessment to ensure that skills and experience of medical physicists are appropriate for the complexity of the tasks to be undertaken. This is particularly important as ROMPs are often engaged in work with significant safety implications for patients, staff, and the public. Radiation protection is one of these areas and ROMPs in all surveyed countries spend at least one hour per week on average on this activity, as can be seen in Table 3a. While the survey did not probe if certification was actually required in a country to practice medical physics, it can be assumed that this is likely to become the norm in the future.

In the context of certification and credentialing, professional organisations play an essential role. As such it is good to see in Table 4 that most countries/territories have a professional association that represents medical physicists and, at least in principle, could oversee a certification scheme. International organisations such as IOMP and AFOMP can facilitate communication between these organisations, possibly help with mutual recognition, and further assist in defining standards to assess medical physics practice.

Finally, it is important to note that CPD is an integral part of most certification procedures [9, 10]. In a fast-changing technological environment such as radiation oncology medical physics, this is particularly essential. As such, education of ROMPs does not end with graduation and there could be opportunities for using educational materials both during the training of students as well as for CPD of experienced ROMPs. This could overcome some of the problems associated with economies of scale in developing educational materials for a small but highly specialised profession.

### Resources and staffing

As can be seen in Table 2a, the number of medical physicists in the region has increased by 29% since 2008. This increase applies to virtually all countries with a notable exception of a few smaller workforces. However, the number of megavoltage treatment units in the region has also increased significantly since 2008 and therefore the ratio of machines per ROMP has only decreased slightly as can be seen in Table 2b. In 2009, AFOMP published its recommendations for staffing levels for medical physicists [11]. Given the variation in practice, it is difficult to apply these figures rigorously; however, given the complexity of work, it appears that the present number of ROMPs is still on the low side of these recommendations.

Table 2 also shows that many countries have implemented intensity modulated radiation therapy (IMRT) [12, 13] and image guided radiation therapy (IGRT) [14, 15]. As a matter of fact, these technologies have become so widely used that some respondents did not even mention them in the survey. The availability of complex treatment units such as Cyberknife surgery and helical tomotherapy has also increased substantially from 2008 to 2011. While the availability of equipment does not necessarily indicate its extensive use, the commissioning of equipment - which is independent of workload - is one of the core activities of medical physicists in radiation oncology [16]. Additional tasks such as planning and individual patient quality assurance (QA) will increase with clinical utilisation, increasing the workload as the added complexity presents additional risks and thus requirements for QA [17, 18].

Table 4 shows that access to the Internet is now available virtually everywhere. This provides significant opportunities for the future as the Internet has become an essential tool for information exchange and access to resources for medical physicists. On the other hand, the availability of specialised dosimetric equipment has not improved dramatically since 2008. Given the increasing complexity of the equipment in most countries, this is of considerable concern.

### Typical tasks and workloads for ROMPs

As can be seen in Table 3a, most of the workload for ROMPs is clinical with treatment planning being the predominant role in most countries. As such, it is fair to say that ROMPs are members of the clinical team directly involved in patient care (albeit not always with patient contact). However, the survey shows that ROMPs also spent a significant amount of time on a large variety of duties including provision of information technology services and radiation protection. The results of this part of the survey illustrate a shortcoming of the simple questionnaire as the large variety of work practices had to be compressed into few categories. This will result in some variation in the interpretation of the answers.

However, there is no doubt that there is significant breadth in the work of medical physicists. This is also illustrated in their involvement in research and teaching, as shown in Table 3b. As can be seen, many physicists are involved in education of other professionals in the hospital environment. This confirms the importance of medical physics concepts but also exposes a shortcoming as most medical physics training does not include teaching and communication skills. Similarly, future training requirements would also need to consider research and professional ethics [19].

Table 3b also summarises the results of the questions regarding overtime. Given the nature of medical physics work, it is common that out-of-hours work is required. This is often in the form of overtime that extends work hours beyond normal working hours. As Table 3b shows, ROMPs in most countries are required to perform overtime work. It is concerning that this is expected but often not adequately compensated. The routine requirement for overtime work also confirms the fact that staffing levels are typically less than adequate for all the tasks required of ROMPs.

### Status and job satisfaction

The perceived high workload is also reflected in Table 5. Most respondents felt that the workload for medical physicists is too high. From the simple questions in the present survey, it is difficult to compare the results directly with 2008. However, it appears that workloads have increased while the professional recognition and remuneration has stayed more or less constant. In any case, it appears that the disparities between countries have not substantially reduced between 2008 and 2011.

### Limitations of the survey

Any survey conducted with individuals representing whole countries and territories has limitations as many countries have complex healthcare systems with a large diversity of tasks and practices. A particular concern is the availability of public and private facilities in most countries that often serve different patient groups and may have considerably different equipment and work practices. The fact that only one person completed the survey in each country will introduce some bias to the results; however, the personal contact amongst the authors ensures that the response rate can be as high as it has been.

Another limitation is the fact that work practices of medical physicists in radiation oncology are different in different countries, as can be seen in Table 3. Therefore some tasks may actually be taken up by other professionals and possibly technicians. While this is not accounted for in the survey, it can be assumed that the physicists will also take on other responsibilities that are not part of their core duties. In assessing staff numbers per machine or population as in Table 2, it was assumed that there is a balance between delegated and newly acquired tasks.

## Conclusion

As organisations such as the ILO start to recognise medical physics as a profession, it is evident that, despite some encouraging signs, there is still a lot of work required towards establishing an adequately trained and resourced medical physics workforce in the Asia Pacific region. The significant increase in the number of ROMPs in the region between 2008 and 2011 is matched by similar increases in radiation oncology equipment and complexity of treatment approaches. As further increases in the use of radiation for cancer treatment can be expected, it will be important to continue also the growth of the medical physics profession.

## Appendix 1: Acronyms for Professional Associations

AAPM: American Association of Physicists in Medicine

ACPSEM: Australasian College of Physical Scientists and Engineers in Medicine

AFOMP: Asia-Oceania Federation of Organizations for Medical Physics

AMPI: Association of Medical Physicists of India

ARPS: Australasian Radiation Protection Society

CSMP: Chinese Society of Medical Physics

CSMPT: Chinese Society of Medical Physics, Taipei

CSRO: Chinese Society of Radiation Oncology

CSTRO: Chinese Society for Therapeutic Radiology and Oncology

HFMBI: Indonesian Medical Physics and Biophysics Association

HKAMP: Hong Kong Association of Medical Physics

IKAFMI: Indonesian Medical Physics Association

IOMP: International Organization for Medical Physics

IPEM: Institute of Physics and Engineering in Medicine

JRS: Japan Radiological Society

JSMP: Japan Society of Medical Physics

JSRT: Japanese Society of Radiological Technology

KSMP: Korean Society of Medical Physics

MAMP: Malaysian Association of Medical Physics

MIP/MP: Malaysian Institute of Physics, Medical Physics Subgroup

NAMP: Nepalese Association of Medical Physicists

PARP: Philippine Association for Radiation Protection

POMP: Pakistan Organization of Medical Physicists

POMP: Philippine Organization of Medical Physicists

PSC: Pakistan Society of Cancer

PSCO: Pakistan Society of Clinical Oncology

PSNM: Pakistan Society of Nuclear Medicine

RSP: Radiological Society of Pakistan

SLMPA: Sri Lanka Medical Physics Association

SMP: Society of Medical Physicists (Singapore)

TMPS: Thai Medical Physicists Society

VAMP: Vietnam Association for Medical Physics
